# Communicable disease among people experiencing homelessness in California

**DOI:** 10.1017/S0950268820000722

**Published:** 2020-03-30

**Authors:** C. Y. Liu, S. J. Chai, J. P. Watt

**Affiliations:** 1 Kaiser Permanente San Francisco/University of California San Francisco Internal Medicine/Preventive Medicine Residency, San Francisco, USA; 2 Division of Communicable Disease Control, California Department of Public Health, Richmond, California, USA; 3 Career Epidemiology Field Officer Program, Division of State and Local Readiness, Center for Preparedness and Response, Centers for Disease Control and Prevention, Atlanta, Georgia, USA

**Keywords:** Infectious disease, people experiencing homelessness, public health, vulnerable populations

## Abstract

California has a large population of people experiencing homelessness (PEH) that is characterised by a high proportion of people who are unsheltered and chronically homeless. PEH are at increased risk of communicable diseases due to multiple, intersecting factors, including increased exposures, comorbid conditions including substance use disorder and mental illness and lack of access to hygiene and healthcare facilities. Data available for several communicable diseases show that PEH in California experiences an increased burden of communicable diseases compared to people not experiencing homelessness. Public health agencies face unique challenges in serving this population. Efforts to reduce homelessness, increase access to health care for PEH, enhance data availability and strengthen partnerships among agencies serving PEH can help reduce the disparity in communicable disease burden faced by PEH.

## Homelessness in the USA and in California

The definition of a person experiencing homelessness (PEH) differs by government agency. The US Department of Health and Human Services defines a homeless individual as one who ‘lacks housing’, which includes ‘an individual whose primary residence during the night is a supervised public or private facility that provides temporary living accommodations and an individual who is a resident in transitional housing’ [1]. The US Department of Housing and Urban Development (HUD) expands the definition to include people living in unstable housing arrangements such as motels, personal vehicles, and tents [2]. Causes of homelessness include lack of affordable housing, unemployment, poverty, and low wages [3].

Measuring the number of PEH is challenging, as housing status can fluctuate over time. One method of measurement is an unduplicated one-night estimate of sheltered and unsheltered individuals who are experiencing homelessness. In the USA, this is conducted yearly during the last week in January by local planning bodies [4]. In 2018, this measurement identified 553 000 PEH in the USA, of which 180 000 (33%) were individuals who are part of families with children. Of the total, 65% were sheltered (living in shelters or motels) and 60% were men. [4] From 2010 to 2018, the number of PEH declined by 13.2% nationally, but increased by 5.3% in California.

**Fig. 1.**
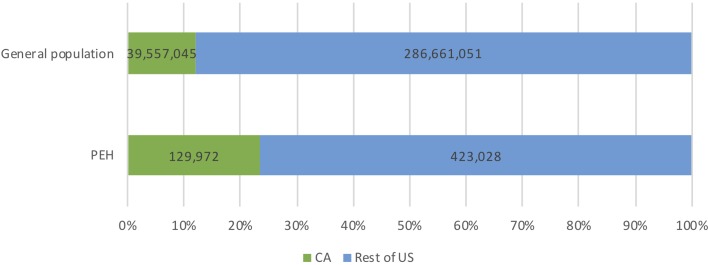
California population of persons experiencing homelessness (PEH).

**Fig. 2.**
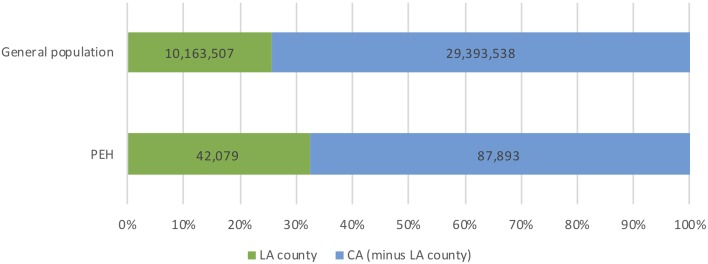
Los Angeles County population of persons experiencing homelessness (PEH).

**Fig. 3.**
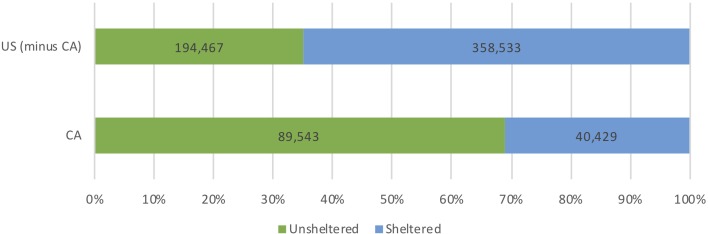
Unsheltered *vs*. sheltered status among persons experiencing homelessness in California and the USA.

California's population of PEH is distinctive due to its size, the proportion of unsheltered individuals and proportion of chronically homeless individuals (defined as a person who is continuously homeless for greater than 1 year, or greater than four episodes of homelessness in 3 years [5]). In 2018, California accounted for 12% of the US population (40 million) [6] and 24% of the total population of PEH (129 972, or 0.3% of California's population) [4] (Fig. 1). Los Angeles County, California's largest county by population, accounts for 25% of California's total population and 32% of California's homeless population (42 079) (Fig. 2). Nearly half (47%) of the US unsheltered homeless population lives in California and more than two-thirds (69%) of California's homeless population is unsheltered (Fig. 3). Finally, 32 668 chronically homeless individuals live in California, representing 25% of all PEH in California and 37% of the US population of chronically homeless individuals [4].

Mortality is increased among PEH compared to the general population, especially among younger people and women [7]. A study of homeless youth (15–24 years) in San Francisco reported an age, race and gender-adjusted standardised mortality ratio of 10.6 (95% CI (5.3–18.9)) compared with California's general youth population [8]. Individuals who are unsheltered or chronically homeless have further increased risk of mortality [9]. Unsheltered PEH has a 2.7-fold increased risk of mortality compared to those who are sheltered [10]. Increased mortality is related to a number of causes including poisoning (medication and illicit substances), suicides, accidents, heart disease, and infections. Increased infection-specific mortality among PEH has been documented, although data are limited because of the difficulties in capturing information on PEH. Studies of human immunodeficiency virus (HIV) infection and tuberculosis (TB) among PEH come from one urban centre or from outside the US [7, 11]. HIV was the leading cause of death for PEH living in Boston during 1988–1993; the leading cause of death shifted to drug overdose and substance use disorders during 2003–2008 [11]. Among PEH diagnosed with TB in Toronto, Canada during 1998–2007, 19% died within 12 months of diagnosis compared to 7.4% of all persons diagnosed with TB [12].

The overall physical and emotional health status of PEH is worse compared to the general population and PEH suffer from a disproportionate burden of communicable diseases compared with the general population [7]. A 2012 systematic review reported an increased prevalence of HIV infection, hepatitis C virus (HCV) infection and TB prevalence among PEH across studies in multiple countries. Among US studies, the prevalence of TB among PEH was 46.7–461.2 times higher compared to the general population. Hepatitis C prevalence was 5.2–17.6 times greater than the general population and HIV infection prevalence was similar to the general population in some studies, or up to 42 times greater. There was significant heterogeneity in these studies, most likely related to differences in population sampling. All studies sampled individuals who engaged in social services, such as shelters or soup kitchens; however, these services differ substantially by location. In addition, for TB, differences in the use of diagnostic testing contributed to differences in prevalence estimates [13].

## Homelessness and communicable disease risk

PEH are at increased risk of communicable diseases due to increased exposures and greater susceptibility to illness (Table 1).

**Table 1.** Major risk factors and communicable diseases identified among people experiencing homelessness

Risk factor	Modes of disease transmission	Communicable disease (examples)
Inadequate access to personal hygiene		
Handwashing and toilet facilities	Fecal-oral	Hepatitis A Shigella Norovirus
Bathing and skin care	Direct inoculation	Skin and soft tissue infections (SSTIs) Group A streptococcal infections
Laundry	Ectoparasite infestations Vector-borne illnesses	Lice Scabies Bed bugs *Bartonella quintana* (louse-borne)
Inadequate access to resting places		
Pressure injury from lying on hard surfaces	Direct contact	SSTIs
Lower extremity stasis dermatitis from lack of places to lie flat	Direct contact	SSTIs
Congregate settings and increased exposures (shelters, tent dwellings)	Droplet Airborne Direct contact Fomites	Norovirus Influenza Tuberculosis Hepatitis A
Exposure to disease vectors	Vector-borne	Mosquito-borne illnesses, Typhus (flea-borne)
Behavioral risks		
Exchange of sex for money Sex while high Sexual assault	Sexual contact	Syphilis Gonorrhea Chlamydia HIV Hepatitis B
Comorbid medical conditions		
Substance abuse, including alcohol, intravenous drug use	Blood-borne Skin disruption	HIV Hepatitis A Hepatitis B Hepatitis C Invasive group A streptococcal infections Methicillin resistant *Staphylococcus aureus*
Mental illness	Increase risk behavior Decreased self-care Delays in care	May exacerbate multiple conditions
Limited access to healthcare		
Limited preventive services	Decreased vaccinations Increased vulnerability to infections	Shingles Pneumonia Hepatitis A
Poorly controlled chronic conditions	Decreased immunity Increased vulnerability to infections	SSTIs
Low health literacy	Delays in care	Pneumonia requiring hospitalisation due to late presentation
Limited medical care	Decreased secondary and tertiary prevention Lack of treatment due to inability to find affected individuals	Severe sequelae of minor medical issues. For example, septic shock from cellulitis related to infected wounds from venous stasis dermatitis.

PEH living in crowded conditions such as shelters have greater exposure to pathogens, which increases the risk of TB [14] and diarrheal illnesses [15]. Lack of access to hygiene facilities also increases exposures to pathogens transmitted via faecal-oral transmission such as hepatitis A [16] and may increase exposure to vectors such as lice, which can transmit *Bartonella quintana* infections [17]. Inadequate hygiene facilities may also worsen minor wounds due to lack of adequate skin care [18, 19]. PEH may also have comorbid substance use disorders or engage in risky sexual behaviour, which can increase the risk of acquisition of infections with pathogens such as HIV, HCV, or HAV [7]. Examples of high-risk sexual behaviour include sex while intoxicated or sex in exchange for goods and services such as money, shelter, or drugs. [20]

Inadequate living situations can cause physical injuries that increase susceptibility to infections. For example, a wheelchair-bound person may develop sacral pressure injuries due to lack of an adequate place to lie down. Treatment for these wounds can be delayed due to lack of access to care, which can result in serious life-threatening infection requiring hospital admission. Increased susceptibility to infection can also be the result of poorly controlled comorbid health conditions, such as diabetes. All these environmental and host-specific risks are interrelated and may have multiplicative effects, leading to a substantial increase in the risk of disease.

## Recent examples from California: communicable disease among PEH

Accurate measurement of communicable disease burden among California's population of PEH is challenging. Many communicable diseases linked to homelessness are not reportable conditions (e.g., norovirus illness). Housing status is not collected by routine surveillance systems for all communicable diseases. Nevertheless, data are available from routine surveillance at California Department of Public Health (CDPH) for several communicable diseases that illustrate the disproportionate burden of communicable diseases among PEH in California (Table 2). We summarise examples of communicable diseases among PEH in California to describe transmission dynamics, the risks encountered by PEH and the public health response. The selected examples represent common communicable diseases with available surveillance or cross-sectional data. Other diseases that disproportionately affect PEH that are not presented include louse-transmitted *Bartonella quintana* infection, which has been previously described in a case series outside of the USA [21].

**Table 2.** Recent reports describing housing status and major communicable diseases in California

Category (reference)	People experiencing homelessness *N*	Homeless per cent %	People not experiencing homelessness *N*	Non-homeless per cent %	Total population n	Data type	Location
California population [4, 6]	129 972	0.3	39 427 073	99.7	39 557 045	Homeless point in time count, Census bureau	All California
Hepatitis A Outbreak, 2017–2018 [23]	372	52.6	335	47.4	707	Outbreak investigation	All California (84% of cases from San Diego County)
Invasive Group A Streptococcal Infections, 2010–2017 [50]	299	44.4	374	55.6	673	CDC Active Bacterial Core surveillance (ABCs)	San Francisco County
Hepatitis C 2016–2018, [67]	680	32.9	1384	67.1	2064	CDPH linkage to care demonstration project	San Francisco County, San Diego County, Los Angeles County, San Luis Obispo County, and Monterey County
Primary and Secondary Syphilis (Female), 2017–2018 [38]	207	22.0	735	78.0	942	Surveillance	California Project Area (all California counties except Los Angeles County and San Francisco County)
Primary and Secondary Syphilis (Male), 2017–2018 [38]	275	6.6	3902	93.4	4177	Surveillance	California Project Area
Tuberculosis, 2017 [55]	106	5.4	1873	94.6	1979	Surveillance	All California
New HIV diagnoses within the last 12 months, 2012–2017 [58]	378	1.3	29 826	98.7	30 204	Surveillance	All California

Data are routine disease monitoring data unless otherwise noted.

## Hepatitis A

Hepatitis A, caused by the hepatitis A virus (HAV), usually causes a mild, self-limiting illness for which supportive measures are the standard of care [16]. Community-based transmission is now uncommon in the US and a rapid decrease in hepatitis A incidence after the introduction of hepatitis A vaccination in 1996 suggests that immunisation played an important role [16]. However, since 2017, the Centres for Disease Control (CDC) has reported outbreaks of hepatitis A in 30 states including California among people who use drugs, people who are experiencing unstable housing or homelessness and men who have sex with men [22]. In California, the hepatitis A vaccine is universally recommended for children and adults at increased risk of infection as specified by the Advisory Committee on Immunisation Practices (ACIP). The California Department of Public Health and local health departments also use the hepatitis A vaccine to respond to hepatitis A outbreaks.

During 2016–2018, California experienced a large hepatitis A outbreak among PEH that was concentrated in San Diego County. This outbreak was the largest hepatitis A outbreak in California since the introduction of hepatitis A vaccination. Statewide, a total of 708 outbreak-related hepatitis A cases were reported, with 465 hospitalisations (66%) and 21 deaths (3%). More than half of cases (52.6%, *n* = 372) were in PEH. The outbreak did not involve the general population and predominantly affected PEH and/or who were using drugs. Of the PEH who developed hepatitis A, more than two-thirds (71.6%, *n* = 263) also reported drug use. This outbreak was notable for the high rate of hospitalisation and death [23]. For comparison, a multistate outbreak of hepatitis A tied to consumption of frozen strawberries in 2016 was linked to 143 cases, 56 hospitalisations (39%) and no deaths [24].

Several factors drove the California outbreak of hepatitis A among PEH, including underlying risk factors, environmental sanitation, and vaccine acceptability and access. PEH were at risk for developing hepatitis A due to exposure to unsanitary conditions and concomitant drug use. PEH were at increased risk of severe disease due to underlying alcohol use disorder and chronic HCV infection. Of people with outbreak-associated hepatitis A, 17% were positive for either HCV antibody (anti-HCV) indicating past or current infection, or HCV RNA indicating current infection. Similarly, the prevalence was high in three other states that experienced hepatitis A outbreaks that affected PEH and people who use drugs in 2017: 49% (29/59) in Kentucky, 26% (165/632) in Michigan and 21% (31/148) in Utah [25].

In the general population, the prevalence of anti-HCV and HCV RNA positivity in the USA is 1.7% and 1%, respectively [26]. Hepatitis A outbreaks are difficult to control due to the long incubation interval, long period of infectivity and a significant proportion of asymptomatic infections. Public health agencies in California used three population-level strategies to respond to the outbreak of hepatitis A among PEH: vaccination, sanitation, and education. Vaccination efforts targeted at-risk individuals (PEH or persons using drugs and living in unstable housing). During this outbreak, the CDPH expanded criteria for hepatitis A vaccination beyond ACIP recommendations, which at the time did not include PEH, to include PEH [27]. Reaching at-risk individuals required partnerships with local organisations, since many people lived in less accessible locations and experienced a lack of trust in the healthcare system. For example, San Diego County public health nurses worked with staff from homeless service providers or law enforcement to form ‘foot teams’ and reach at-risk individuals in riverbeds, canyons, ravines, parks, and urban encampments. In San Diego County alone, 121 921 hepatitis A vaccinations were delivered during this outbreak [28]. To support vaccination efforts, California Governor Jerry Brown declared a state of emergency in October 2017, which enabled the state to ensure adequate vaccine supply for outbreak response [29]. Sanitation interventions included increasing access to bathrooms by extending hours of bathroom facilities and setting up portable toilets and handwashing stations. Education interventions were aimed at high-risk populations, health care providers and the general public. Specific tools included pamphlets explaining the role of hygiene in interrupting disease transmission and hygiene kits. Collaboration with local media outlets was critical for the dissemination of these messages.

The California hepatitis A outbreak ended in 2018, but other outbreaks are ongoing in the USA. In February 2019, ACIP updated its recommendations for hepatitis A vaccination to include PEH [30]. ACIP recommends routine hepatitis A vaccination in children [31] and adults with risk factors, including homelessness, chronic liver disease, clotting factor disorders, men who have sex with men, work with HAV in a research laboratory, travel in countries with high or intermediate endemic hepatitis A, or close personal contact of an international adoptee [32]. Delivering the hepatitis A vaccine to this population remains a challenge, particularly given the existing challenges PEH face when accessing medical care. One potential delivery mechanism is via requirements imposed by California Senate Bill 1152 (SB1152), which requires hospitals to offer appropriate vaccinations to homeless patients on inpatient or emergency room discharge [33].

## Syphilis

Syphilis, a sexually transmitted genital ulcerative disease caused by the bacterium *Treponema pallidum*, is associated with significant adverse consequences if left untreated with progression into secondary and tertiary syphilis. The incidence of syphilis has increased nationally and in California despite the usual efforts of public health departments, which includes contact tracing, screening, and treatment with penicillin [34,35]. In 2017, the incidence rate of primary and secondary syphilis was 16.8 cases per 100 000 Californians, up from 1.0 cases per 100 000 in 2000 [36]. Among women, cases increased by 600% during 2012–2017 [37]. Homelessness disproportionately affects people with syphilis: 7% of men and 21% of women in California with a new diagnosis of syphilis during 2017–2018 [38]. In comparison, PEH represent 0.3% of California's total population. The significant burden of homelessness in women is particularly concerning in light of the rise in congenital syphilis (CS) cases in California. Previous work in other states on mothers of infants with CS indicates that unstable housing status is associated with increased risk of CS [39, 40]. Outbreaks of syphilis have also occurred among PEH. In 2018, Sonoma County identified a cluster of syphilis cases among homeless individuals living in encampments [41]. In response, Sonoma County Department of Public Health initiated targeted screening efforts both in the field and through providers serving persons at increased risk in order to identify additional cases and provide treatment.

The association between homelessness and syphilis may be related to multiple intersecting factors. For example, substance use disorders, which are common among PEH [7] and sex while high has been associated with increased risk of syphilis in women [42]. Lack of access to care and inadequate screening and treatment, which are also common among PEH [7], may result in continued spread within sexual networks. PEH may exchange sex for services or goods [43] and these activities increase the risk for sexually transmitted infections, such as syphilis, due to a large number of partners and inability to negotiate consistent condom use [44]. Current efforts to address the rising rates of syphilis in the general population include expansion of populations screened for syphilis in screening guidelines for sexually transmitted infections and targeted screening interventions. Current CDC guidelines recommend syphilis screening for all pregnant women, sexually active MSM, men at increased risk and HIV positive individuals [45]. One example of a targeted screening intervention that could reduce the burden of syphilis is to increase screening in the correctional system. In California state prisons all inmates are screened for syphilis and treated if syphilis is diagnosed. The California Department of Public Health encourages providers serving PEH, including hospital emergency departments, to provide sexually transmitted disease screening for PEH.

## Invasive group A streptococcal infections

Group A *Streptococcus* (GAS) bacteria cause a variety of minor infections, including pharyngitis and skin infections. Invasive group A streptococcal infection (iGAS) is defined as infection with GAS isolation from a normally sterile site (blood) or wound with necrotizing fasciitis or streptococcal toxic shock syndrome [46]. The CDC tracks cases through the Active Bacterial Core surveillance (ABCs) system, which collects data on several pathogens, including Group A *Streptococcus,* from sites in 10 states, including three counties in California [47]. iGAS is associated with increased mortality (case fatality ratio 11.7%). Factors independently associated with death included increasing age and underlying chronic illness [48]. Outbreaks of iGAS have been described among individuals experiencing homelessness and/or individuals who use injection drugs in Europe [49], Canada [50] and the USA [51].

While iGAS is not routinely reportable in California, a study of 673 iGAS cases in San Francisco during 2010–2017 tracked by the ABCs system found that 34% of cases were among PEH. The incidence of iGAS increased significantly from 300 in 2010–2014 to 547 in 2017 per 100 000 population among PEH and from 5 in 2010–2013 to 9.3 in 2017 per 100 000 among people not experiencing homelessness. The iGAS incidence was greater among PEH compared to people not experiencing homelessness due to multiple risk factors, including substance use disorders and barriers to appropriate skin care [46]. Individuals who use injection drugs may develop infections from contaminated drugs, needles, or drug paraphernalia. Recent work from New Mexico evaluating risk factors for iGAS among PEH suggested that skin breakdown and barriers to appropriate skin care may increase the risk of iGAS [52]. Collaboration with organisations serving PEH and people who inject drugs, such as needle exchange organisations, is important for preventing infections in this population and for providing care to limit the severity of wounds that have already developed. For example, existing needle exchange services not only provide needles and syringes but also educate people on appropriate skin care and harm reduction methods [53].

## Tuberculosis

TB is caused by *Mycobacterium tuberculosis* and commonly causes pulmonary infections or asymptomatic latent infection. The TB incidence rate nationally among people experiencing homeless was estimated from 36 to 47 cases per 100 000 population in 2006–2010 [54], compared to 2.8 per 100 000 population in the general population in 2017 [55]. While TB is more common in PEH than the general population, multi-drug resistant (MDR) TB was less common. MDR infections affected 1% of PEH with TB [54], compared to 1.9% of all people diagnosed with TB in the US in 2018 [56]. Among patients with TB in 2017, 5.6% of TB cases in California reported homelessness, compared to 4.6% nationally [55]. In California, using the point in time estimate of the population of PEH, the incidence rate of TB among PEH in 2017 was 94 per 100 000 population compared to 4.7 per 100 000 population in the general population.

Los Angeles County has been experiencing a large TB outbreak among PEH since 2013. Efforts to address TB among PEH in Los Angeles County have had some success; the percentage of TB cases reporting homelessness declined from 10.1% in 2013 to 7.6% in 2016 [57]. Using the point in time estimate for the denominator, the incidence of TB among PEH decreased from 132 per 100 000 population in 2013 to 89 per 100 000 population in 2016. Los Angeles County Department of Public Health has collaborated with medical providers serving PEH and shelter sites to provide TB symptom screening at shelter entry, targeted testing, and TB treatment. The Agency also has a programme to temporarily house high-risk individuals receiving latent TB infection treatment [57].

There remain significant challenges to the treatment of active TB and latent TB infection among PEH. First, current information systems may not accurately reflect a person's true housing status, especially for people with unstable housing who may be intermittently homeless and housed. Therefore, reaching exposed individuals remains a challenge as contact tracing is difficult due to the lack of or transient living addresses of PEH. CDPH has successfully used external data from the HUD's Homeless Management Information System (HMIS) to assist with locating potential contacts. However, this information is imperfect, as people change locations over time. Second, adherence to treatment is a major challenge given the extended duration of therapy. For patients with latent TB infection, short-course therapy with rifapentine can help address issues with non-adherence due to length of therapy. Finally, exposures in congregate living situations such as homeless shelters may increase the risk of TB exposure among PEH.

## HIV infection

HIV infection is no longer the leading cause of death among PEH [11]. While no recent outbreaks have been reported in California, PEH experience a disproportionate burden of new HIV infection diagnoses. In California, from 2012 to 2017 across all counties, 1.3% (378/30 204) of new cases of HIV infection were in people identified as experiencing homelessness or unstable housing based on home address [58]. During the same period, 1% of people living with HIV were homeless or unstably housed [59]. Data from the Medical Monitoring Project (MMP) in California, which includes data for all counties except for San Francisco and Los Angeles, estimates that 10.8% (95% CI 6.7–14.9%) of California adults living with HIV reported homelessness during the 12 months before the interview [60]. The difference in the estimates may be related to the differences in data collection. Surveillance data include all counties in California, while the MMP includes all counties except for San Francisco and Los Angeles. Surveillance data measure housing status at a point in time based on the classification of a patient's home address, while the MMP asks people directly about their housing status over the past year. In 2017, based on the point in time population of PEH, the incidence rate of newly diagnosed HIV infection among PEH in California was nearly fivefold that of the general population: 56 cases per 100 000 population among PEH compared to 12 cases per 100 000 population in the general population. In San Francisco, 14% of new HIV infection cases were among PEH, and PEH had delays in time to viral suppression compared to those who were not homeless [61].

Previous research demonstrates that housing decreases mortality among people living with HIV [62]. Several programmes help PEH who have HIV infection to find housing, including the Ryan White programme [63] and the Housing Opportunities for Persons with AIDS Programme administered by the US Department of HUD [64]. Collaboration with local governments and organisations is key to meeting the needs of this population. In San Francisco, the Getting to Zero initiative advocates for people living with HIV to be prioritised for housing [65]. In 2018, San Francisco Department of Public Health received CDC funding for Project OPT-IN, a 4-year demonstration project to improve HIV treatment outcomes among vulnerable populations, including PEH. Proposed services include homeless outreach and intensive care management. [66]

## Hepatitis C

Hepatitis C disproportionately affects PEH [13]. In California, housing status is not routinely collected by the hepatitis C disease monitoring system. Information on hepatitis C and housing status comes from California Department of Public Health's hepatitis C testing and linkage to care demonstration projects in four sites serving five counties (San Luis Obispo, Monterey, San Francisco, San Diego and Los Angeles), which found that out of 2064 individuals who newly tested positive for HCV RNA between 1 March 2016 and 30 June 2019, 32.9% (680) were known to be unstably housed [67]. While these data are unlikely to be generalisable to all persons testing positive for HCV since they specifically targeted PEH and people who use injection drugs, they suggest that homelessness may be common among persons infected with HCV in California. Unstable housing is a common barrier to hepatitis C treatment. Collaboration with local organisations and governments is important to find innovative ways to reach PEH who have hepatitis C infection. This is already at work in San Francisco: the End Hep C SF campaign aims to ensure access to hepatitis C treatment for all people, including PEH. This collaborative effort involves the San Francisco Department of Public Health, community clinics and homeless shelters that offer on-site hepatitis C treatment. [68]

## Policy implications

There are multiple potential factors that increase the risk of homelessness and potential areas in which to intervene.

*Housing*: Preventing homelessness and providing housing for PEH can eliminate many of the risks that PEH face. Housing decreases mortality among people with HIV [62] and programs now exist to provide housing for this population. Limited evidence suggests that housing will be beneficial for other groups as well: among chronically ill homeless adults, permanent supportive housing was associated with decreased mortality. Only 2% of the housed individuals died of infectious causes compared to 13% of unhoused individuals [69]. Permanent supportive housing programmes provide a suite of healthcare and social services to help individuals stay healthy and housed [69]. For example, treatment of comorbid substance use disorders and mental illness can help decrease the risk of communicable diseases and increase a person's capacity for self-care. [7]

States have experimented with using Medicaid dollars to pay for housing [5], but addressing the root causes of homelessness, including addressing issues of affordable housing, are fundamental for mitigating the risks to health among PEH.

*Improving care for PEH*: Efforts to improve access to healthcare for PEH could prevent or mitigate illness in more individuals. One strategy is recent California legislation (SB1152) which mandates hospitals to offer appropriate vaccinations and coordination of care for homeless patients on hospital or emergency room discharge. Another strategy is the California Whole Person Care pilot program that is intended to enhance coordination of health and social services for vulnerable populations including PEH.

*Improving information systems*: Reaching exposed or at-risk individuals remains a major challenge, especially when these individuals do not have permanent contact information. Use of information systems like the California Immunisation Registry (CAIR) [70] and the HUD's HMIS [71] could support improved information sharing between different homeless service providers and public health departments to reach people in a timely fashion and provide needed services [72]. The Health Insurance Portability and Accountability Act protects certain health information and may be a barrier for health care systems to collaborate with homeless service providers. [73]

*Strengthening partnerships:* Public health departments must work closely in partnership with local governments and non-governmental organisations to meet the health needs of PEH. One important group of partners are community health centres that receive funding through the federal Health Care for the Homeless program. In the case of the 2017–2018 hepatitis A outbreak, collaboration between the department of public health and local social services agencies was crucial for reaching thousands of at-risk individuals.

## Summary

California has a large and growing population of PEH. Compared to the rest of the country, California's population of PEH are disproportionately unsheltered and chronically homeless. These individuals die prematurely and suffer a greater burden of illness, including communicable diseases such as HIV, hepatitis A, hepatitis C and TB. PEH are especially vulnerable due to the intersection of multiple risks, including increased exposure to pathogens, decreased immunity and decreased access to healthcare and services that would mitigate the severity of illness.

There are several important limitations to the available data on housing status and communicable disease risk. First, disease incidence among PEH may be overestimated due to undercounting of the true population of the PEH. Calculations of disease incidence rely on HUD's yearly point-in-time counts, which may fail to include people in hard to reach locations (riverbeds, remote encampments) and people intermittently cycling between homelessness and unstable temporary housing arrangements. Second, case burdens may appear elevated due to enhanced screening among PEH. For example, targeted syphilis screening among PEH in Sonoma County may partly explain the elevated proportion of PEH among people with syphilis. Conversely, it also may be the case that PEH are not properly identified due to lack of medical care or inability to trace contacts due to lack of information. Finally, housing status is inconsistently collected across disease categories and a significant amount of housing information is missing, which can affect disease incidence estimates in either direction.

Efforts to address communicable disease risk in this population will depend on innovative strategies to overcome the unique challenges in reaching people and providing adequate treatment, as well as strategies to address fundamental causes related to access to affordable housing and treatment for comorbid conditions including substance use disorder and mental illness. While some of the root causes of homelessness lie outside the purview of public health agencies, there are opportunities for public health to support both primary and secondary prevention of communicable disease issues in PEH. Examples of primary prevention efforts by public health include immunisation, promotion of harm reduction methods including needle exchange and efforts to increase access to mental health services, substance use treatment and supportive services for PEH. Examples of potential secondary prevention interventions include supporting access to culturally competent clinical and other supportive services to reduce the complications of infections once they occur.
